# Estradiol-Induced Epigenetically Mediated Mechanisms and Regulation of Gene Expression

**DOI:** 10.3390/ijms21093177

**Published:** 2020-04-30

**Authors:** Tamás Kovács, Edina Szabó-Meleg, István M. Ábrahám

**Affiliations:** 1 Molecular Neuroendocrinology Research Group, Institute of Physiology, Medical School, Centre for Neuroscience, Szentágothai Research Center, University of Pécs, H-7624 Pécs, Hungary; tamas.kovacs@aok.pte.hu; 2 Department of Biophysics, Medical School, University of Pécs, H-7624 Pécs, Hungary; edina.meleg@aok.pte.hu

**Keywords:** estradiol, histone proteins, methylation, demethylation, histone modification enzymes

## Abstract

Gonadal hormone 17β-estradiol (E2) and its receptors are key regulators of gene transcription by binding to estrogen responsive elements in the genome. Besides the classical genomic action, E2 regulates gene transcription via the modification of epigenetic marks on DNA and histone proteins. Depending on the reaction partner, liganded estrogen receptor (ER) promotes DNA methylation at the promoter or enhancer regions. In addition, ERs are important regulators of passive and active DNA demethylation. Furthermore, ERs cooperating with different histone modifying enzymes and chromatin remodeling complexes alter gene transcription. In this review, we survey the basic mechanisms and interactions between estrogen receptors and DNA methylation, demethylation and histone modification processes as well as chromatin remodeling complexes. The particular relevance of these mechanisms to physiological processes in memory formation, embryonic development, spermatogenesis and aging as well as in pathophysiological changes in carcinogenesis is also discussed.

## 1. Introduction

Epigenetics can be simply defined as “heritable changes in genome function that occur without changes in the DNA sequence” [1,2]. The term epigenesis comes from the Greek prefix “epi”, meaning “over”, and genetics, which means all the study of heredity and all different types of work with DNA. The major epigenetic changes are DNA cytosine methylation; the hydroxylation of a methylated cytosine residue (5hmC); and post-translational histone modifications such as acetylation, hydroxylation, phosphorylation and ubiquitination. DNA and histone protein modifications play a crucial role in epigenetic inheritance [3]. Although chromatin remodeling is usually not inherited, it influences gene transcription by changing the accessibility of chromatin to the transcription complexes, resulting in changes in the phenotype of the cells [4]. In addition, the long non-coding, small interfering or micro RNAs and the changes in the chromatin conformation also play a role in epigenetic mechanisms [5,6,7].

The gonadal hormone, 17β-estradiol (E2) influences a wide range of biological phenomena, from fertility to memory formation [8,9,10]. E2 binds to the ligand binding domain (E-domain) of intracellular estrogen receptors (ERα, ERβ). After ligand binding, ERα and ERβ form homodimers and heterodimers [11]. Dimerized ERs, as a ligand-activated transcription factor, interact with the estrogen responsive elements (EREs) on the DNA, in turn inducing or repressing gene transcription [12,13,14]. Besides their classical genomic action on EREs, ERs alter gene expression by methylating the transcription factor binding sites—cytosine and guanine rich regions in the genome, so called CpG islands—in promoter or enhancer regions. E2-mediated processes actively acetylate or methylate the histone proteins [15,16]. Interestingly, E2 is a key component in passive and active DNA demethylation processes both on the DNA and on histone proteins. Moreover, E2 is able to regulate the chromatin’s structure by remodeling chromatin accessibility.

Although knowledge is relatively limited, we make an attempt to highlight aspects of recently acquired insight into the role of E2 in epigenetic mechanisms and potential consequences. Accordingly, in this review, our first goal is to describe the E2-induced DNA and CpG island methylation as well as demethylation processes. Moreover, we discuss how ERs interact with histone modification enzymes and chromatin remodeling complexes. Finally, the physiological and pathophysiological relevance of E2-induced epigenetic alterations will be summarized.

## 2. E2 Alters Gene Transcription via DNA Methylation

In order to understand the mechanism of E2-induced methylation, we first discuss the role of CpG islands. In the methylation process, catalyzed by DNA methyltransferases (DNMTs), a methyl group is transferred from S-adenyl methionine (SAM) to the 5-carbon of a cytosine residue in order to form 5-methylcytosine (5mC) in the CpG island [17]. There are two DNMTs (DNMT1, DNMT3) with distinct functions. DNMT1 is active during DNA replication to copy the DNA methylation pattern from the parental DNA strand [18]. DNMT3, the so called de novo methyltransferase, has three different isoforms, DNMT3a, DNMT3b and DNMT3l. DNMT3a and DNMT3b establish new methylation patterns on unmodified DNA. By contrast, DNMT3l does not bind to DNA but forms a complex with other DNMT3 proteins, methylates cytosines and stimulates their activity [19,20,21]. CpG islands are the typical sites of methylation, with around 1000 bp long evolutionarily conserved DNA sections and promoter regions regulating gene expression and chromatin structure [22,23]. Importantly, the epigenetic modifications of CpG islands alter the patterns of gene expression. When methylation occurs in the promoter region or in the transcription binding sites of a gene, it represses transcriptional activity [24]. However, the mechanism is more complex because methylation has site-specific effects. While the methylation blocks transcription in the transcription starting site, it promotes transcription in the gene body [25,26]. 

E2 initiates a wide range of epigenetic changes including the methylation of the CpG island. In general, ERs bind to the estrogen responsive elements (EREs) in the nucleus and induce gene transcription. However, the genome is more likely to be methylated in the CpG island and less so at ERE sites in breast cancers [27]. For instance, Marques and colleagues demonstrated that ERα methylates the CYP1A1 locus [28] and that ERα leads to the silencing of the progesterone receptor, epoxide hydrolase 2 (Ephx2), lipocalin 2 (LCN2) and interferon α inducible protein 27 (IFI27) genes via CpG island methylation [29,30]. All together, these results suggest that the function of liganded ERs involves the methylation of CpG promoters and gene silencing.

Several experiments demonstrate that E2 alters the mRNA and protein expression of DNMTs. A recent paper showed the importance of octamer binding transcription factor (OCT4) and ERα in ERE-mediated DNMT1 expression [31]. OCT4 does not promote the expression of DNMT1 in ERα-positive breast cancers because ERα occupies the ERE sequence in the promoter region of DNMT1 and inhibits DNMT1 expression [31]. Following E2 microinjection into the dorsal hippocampus, hippocampal DNMT3a and DNMT3b mRNA expression and protein level increased [28,32,33] but DNMT1 expression was not changed [33]. Li and colleagues demonstrated that the DNMT3b protein level is elevated in response to E2 treatment in ERα-positive MCF7 cells [33]. Moreover, E2 treatment exclusively activates DNMT3b in MCF7 cells and methylates the promoter region of ribosomal protein kinase (RSK4). These results demonstrate that E2 treatment exclusively increases DNMT3b expression. Since DNMT3b is a de novo methyl transferase, these results also suggest that ERα has a role in the formation of new DNA methylation and alters the initiation of transcription via DNMT-mediated DNA methylation [33].

**Figure 1:**
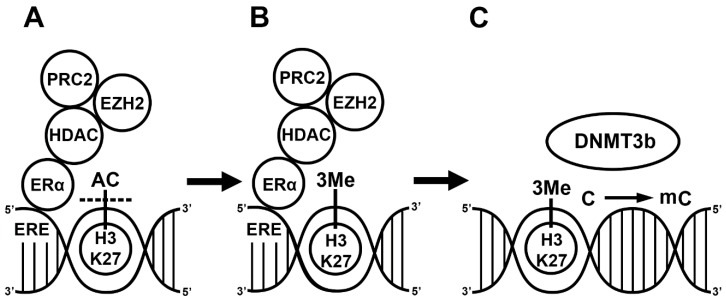
17β-estradiol (E2)-induced methylation mechanism. Liganded estrogen receptor (ER) binds to the estrogen responsive element (ERE) in the genome. Estrogen receptor recruits histone deacetylase 1 (HDAC), polycomb repressive complex 2 (PRC2) and enhancer of zeste homolog 2 (EZH2) (**A**). The HDAC removes acetyl groups from the histone 3’s 27th lysine residue (H3K27). EZH2 places three methyl groups, 3Me, on H3K27 [29] (**B**). DNA methyltransferase 3b (DNMT3b) recognizes the methylated H3K27 and methylates the cytosine (C) in a CpG island (**C**) [44].

Regarding the mechanism, E2 indirectly activates the function of DNMT3b since ERs alone do not bind to any of the DNMT enzymes. First, ERα recruits co-regulators, such as the nuclear receptor interacting protein (NRIP1) [34]; the repressor of estrogen activity (REA) [35], which is a mutant estrogen receptor; and the metastasis-associated factors (MTA1, MTA3) [36,37]. This complex subsequently recruits additional co-repressors such as the histone deacetylase (HDAC1) and polycomb complex 2 (PRC2). The HDAC and enhancer of zeste homolog 2 (EZH2), the enzymatic part of PRC2, first deacetylate the activating histone marks and then place a repressive methyl group on the lysine residues of the histone protein in the nucleosomes of a promoter [38,39]. Although liganded ERα can inhibit the expression of HDAC, E2 induces the expression of the EZH2 gene in rat mammary glands [40,41]. This complex activates DNMT3 in two ways. Firstly, the trimethylation of H3K27 or H3K36 near the CpG islands activates DNMT3a and DNMT3b [42]. Secondly, the PRC2 complex including EZH2 and NurD binds to the DNA methyl transferase complex (DNMT3a, DNMT3b, DNMT3l), and the DNMTs then methylate the CpG island [43,44,45,46]. In summary, liganded ERα plays a role in the regulation of gene transcription by influencing the methylation status of the histone protein and cytosine residues in the CpG islands. ERα recruits co-repressors (HDAC1, PRC2) and different protein complexes (PRC2, NurD) in the methylation process. The interaction between ERα, co-repressors and different protein complexes leads to CpG island methylation in the ERE. The DNMT activation alone is not sufficient, and histone methylation and chromatin remodeling are required to block gene transcription (Figure 1).

## 3. E2-Induced Demethylation via ERs

During the demethylation process, the CpG island loses the methyl group, which leads to the initiation of gene transcription [47]. In contrast to DNA methylation, demethylation is a more complex mechanism. The DNA demethylation process for CpG islands can be either passive or active or a combination of both. Passive DNA demethylation occurs when the newly replicated DNA strand lacks the methylation signal. During normal cell function, passive demethylation takes place when DNMT1 fails to place a methyl group on the newly synthesized strain. During normal cell function, passive demethylation only happens when DNMT1’s function is blocked [31]. In contrast to in the passive process, the 5mC undergoes a very complex chemical modification during active demethylation. The 5mC can be modified in two different ways. The first way is the oxidation of the 5mC into a 5hmC by the ten-eleven translocation enzymes (TETs: TET1, TET2, TET3) [48]. TET2 is different from the other two TETs because it lacks the DNA binding domain [49]. The 5mC is further oxidized to 5-formyl-cytosine (5fmC) and then to 5-carboxyl-cytosine (5caC) [49]. Another way of 5mC residue modification is with activation-induced cytidine deaminase/apolipoprotein B mRNA editing enzyme (AID/APOBEC)-mediated deamination [50,51]. AID/APOBEC deaminates 5mC to form 5-hydroxymethyl-uracyl (5hmU) [52,53]. The TET and the AID/APOBEC modifications are recognized and repaired by the base excision complex (BER), which replaces the modified cytosine residue with a naked cytosine. In addition to its role in the demethylation processes [54], the BER protein is responsible for repairing small damages in the genome during DNA replication [55]. The complex thymine DNA glycosylase (TDG), the other major component of the complex, is essential for the final step of active DNA demethylation [56,57]. The TDG enzyme removes the modified thymine, 5hmU, 5fmC and 5caC and replaces them with cytosine [58].

Previous investigations indicated that CpG islands in breast cancers are more likely to be demethylated in EREs [27]. A great body of evidence suggest that E2 induces gene expression via demethylating promoter or enhancer regions in the genome [27,59,60]. ERα plays a role in passive demethylation, via the ERE-mediated inhibition of DNMT1 expression. Although liganded ERα binds to the ERE in the DNMT1 promoter, it does not initiate gene transcription, rather inhibiting other transcription factors to initiate the gene expression of DNMT1 [31].

**Figure 2:**
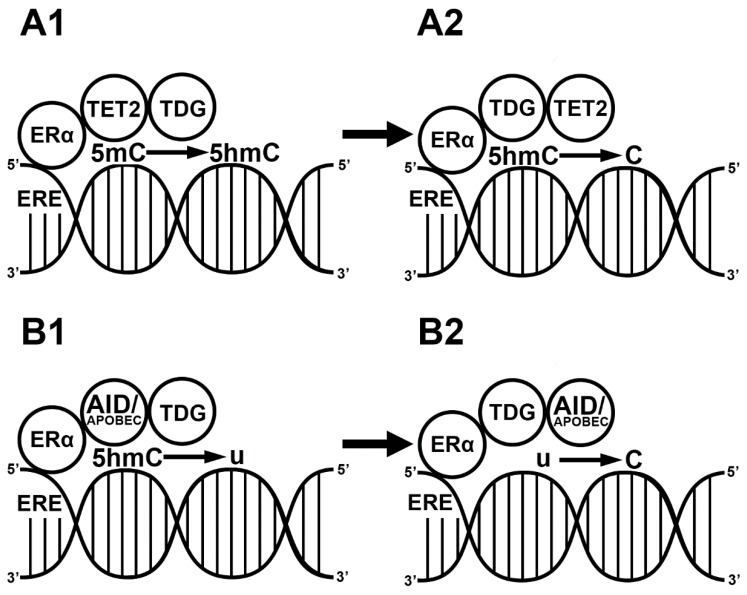
E2-induced demethylation mechanisms. Liganded estrogen receptor (ER) binds the estrogen responsive element (ERE) in the genome. ER recruits ten-eleven translocase 2 (TET2) and thymine-DNA glycosylase (TDG). TET2 hydroxylates the methyl cytosine residue (5mC) into a hydroxymethyl cytosine (5hmC) (**A1**). TDG replaces the 5hmC with a naked cytosine (C) (**A2**). ER binds to EREs in the genome. ER recruits activation-induced cytidine deaminase (AID) and apolipoprotein B mRNA editing enzyme (APOBEC). The ER/AID/APOBEC complex deaminates a previous 5hmC into a uracil (U) (**B1**). TDG replaces the uracil with a naked cytosine (**B2**).

Besides the passive demethylation of CpG islands, E2 also induces active demethylation in two different ways. The first way is mediated via the TET enzymes. The liganded ERα exclusively activates the expression of TET2 by binding to ERE sequences in the promoter region [61] and forming complexes with TET2 and BER. After the modification of the methyl group, the BER complex, through TDG (enzymatic part of BER) and p300 (histone acetyl transferase, E1A-associated protein), replaces it with a naked cytosine residue. In the BER complex, p300 and TDG interact with ERα [62], replace the 5hmC with a naked cytosine and promote gene expression [61]. In addition, E2 also activates the expression of zinc finger proteins such as CXXC4 and CXXC5 in the ERE region. CXXC proteins interact with TET2 in the nucleus and demethylate the cytosine residues. Through the activation of both the TET2 enzyme and CXXC4/CXXC5, E2 is a regulator of the hydroxylation of the methylated CpG islands at enhancer regions [63,64,65] (Figure 2a). The second way of E2-induced active demethylation is 5hmC deamination by AID/APOBEC. The liganded ERα promotes the deamination of methylated cytosine residues by binding to the ERE sequence in the promoter region of AID [66]. Although liganded ERα in the nucleus interacts with APOBEC3B, causing cytosine-to-uracil transition [67], APOBEC is less important than AID in the E2-induced deamination processes. Taken together, E2 plays a critical role in 5hmC deamination because it forms a complex with APOBEC enzymes and activates the expression of AID enzymes [67,68] (Figure 2b).

There are significantly less data available about the role of ERβ in epigenetic processes. However, this limited information suggests that ERβ has a relatively straightforward function. ERβ inhibits the expression of all three DNMT enzymes and thus represses DNA methylation. Since ERβ recruits both TDG and TET to form a complex, it plays a role in the active demethylation processes in similar way to ERα [69,70].

## 4. E2-Induced Histone Modification

Histone proteins are alkaline proteins that constitute the protein part of the nucleosome and play a critical role in the regulation of gene transcription. They are modified post-translationally at different sites, representing an activation or repression mark for transcription factors [71]. Histone 3 (H3) and histone 4 (H4) have long tails, in particular, and are therefore modified most frequently [72]. These modifications influence gene transcription by changing the histone-mediated DNA packaging. The two most common post-translational histone modifications are acetylation and methylation [72].

Histone acetylation is a dynamic epigenetic modification playing a critical role in the regulation of transcription. In this process, an acetyl group from acetyl-CoA is placed on the histone protein. Acetylation generally occurs on the lysine residue and is usually considered to be a transcription activation signal [73]. The liganded ERs play a pivotal role in the mechanism of post-translational histone modifications such as acetylation [74,75]. In order to acetylate the histone proteins, ERα recruits histone acetylases such as p300, which works in complex with the cAMP responsive element binding protein family and the p160 steroid receptor coactivators (SRC1/SRC2/SRC3) [76,77,78]. Guertin and colleagues showed that there is interaction between ERα histone acetyl transferase p300 via SRC proteins at ERE sites within the genome [79]. Furthermore, Frick and colleagues also demonstrated that liganded ERα and ERβ acetylate H3 through the ERK1/2 signal transduction pathway.

The other most common post-translational modification is histone methylation, which is associated with both the activation and repression of gene transcription. For example, the methylation of histone 3 lysine 4 (H3K4) is associated with the initiation of gene transcription, but methylation on histone 3 lysine 27 (H3K27) is a repressive mark [80]. Mixed lineage leukemia genes (MLL1, MLL2, MLL3) are potent histone methyltransferases, and they only methylate H3K4 residues [81]. ERs interact exclusively with MLL2 and place methylation marks on H3K4 residues, which promote gene expression [82]. Besides activation, histone methylation can represent a repressive signal for gene expression. When the H3K27 residue is methylated, it is a repressive signal for gene transcription. ERα interacts with several histone modification enzymes (HDAC, EZH2) and histone modification complexes (NurD, PRC2). These complexes replace the activating acetylation mark with a repressive methyl group on the 27th and 36th lysine residues of H3 [83]. The trimethylated H3K27 and H3K36 are repressive histone marks and therefore inhibit gene transcription [83].

After summarizing these two post-translational modifications, it is worth mentioning that there are proteins that regulate the interaction of ERα and the histone modifying complexes in the nucleus. One of them is the Transcriptional Repressor GATA Binding 1 (TRPS1) gene, which regulates the interaction of ERs with protein complexes in the nucleus. In a recent paper, Serandour and colleagues showed that TRPS1 binds the histone deacetylase complexes, NurD and coREST, both containing histone deacetylases such as HDAC1. Through this process, TRPS1 inhibits the histone deacetylation at ERE sites in the genome [84]. Furthermore, TRPS1 inhibits the E2 binding of liganded ERα to the DNA and ultimately inhibits the expression of different genes [84]. It is tempting to speculate that in the presence of TRPS1, ERα does not form complexes with either histone deacetylases or repressive histone methylases, such that both the post-translational histone modification and DNA methylation functions of ERα are blocked. Further experiments are required to examine the precise role of TRSP1 in E2-induced histone modification.

## 5. E2-Induced Chromatin Remodeling

Chromatin remodeling is a dynamic rearrangement of the chromatin structure from a condensed state to a transcriptionally accessible state. This dynamic change represents a crucial mechanism in epigenetic modification and is carried out by two distinct mechanisms. As discussed above, one mechanism includes post-translational histone modifications, when the histone acetyltransferases, deacetylases and methylases influence the accessibility of the transcription machinery to the genome [85]. The other pathway is regulated by ATP-dependent chromatin remodeling complexes that restructure the nucleosomes. The ATP-dependent chromatin remodelers are grouped into four families: SWItch/Sucrose Non-Fermentable (SWI/SNF), imitation SWI (ISWI), Nucleosome Remodeling Deacetylase (NuRD/Mi-2/CHD), chromatin remodeling INO80 and SWR1 complex (INO80 and the SWR1 complexes belong to one chromatin remodeling family). The different remodelers are similar in their ATPase domains and play crucial roles in distinct biological functions. For example, the ISWI complex is important in proper chromatin assembly after replication. The SWI/SNF and INO80 complexes are involved in the repair of DNA double-stranded breaks as well as in the base excision repair mechanism [86]. The SWI/SNF-related transcriptional activators such as BRG1 (SWI/SNF related-matrix associated-actin dependent-regulator of chromatin-subfamily a-member 4) and BAF57 (SWI/SNF-related-matrix associated, Actin Dependent Regulator of Chromatin, Subfamily E, Member 1) play pivotal roles in the activation of repressed genes and transcription initiation, respectively. The INO80 complex plays a critical role during embryonic development [87]. It was reported that in the embryonic stem cell, INO80 recruits important pluripotency transcription factors such as OCT4, Nanog and SOX2 [87,88].

A recent finding showed that E2 treatment changed the chromatin structure on both large and small scales in the genome [89]. In the E2-induced chromatin remodeling mechanism, the liganded ERα interacts with BRG1 and BAF57 and promotes the activation of the MLL/HAT and p160 histone acetyl transferase, respectively [90,91,92]. The AT-Rich Interaction Domain 1A, B (ARID1A, ARID1B) protein is part of the SWI/SNF complex. The loss of the ARID1A gene leads to the compensatory upregulation of ARID1B. However, this does not rescue the ERα-dependent transcription, which suggests the critical role of the ARID1A protein in ERα-related functions [88].

Although there is no direct interaction with ERα, INO80 stabilizes the ERE sites in the enhancer regions of the gene [93]. Accordingly, INO80 promotes ERα-induced gene transcription [93]. As discussed earlier, ERα can interact with MTA1 and HDAC1 of the NurD complex. Besides its role in CpG island methylation, ERα may alter chromatin remodeling via NurD. However, ERα inhibits the expression of MTA1 and HDAC1 [94,95]. These interactions lead to DNA methylation.

## 6. Key Players in ERα-Mediated Epigenetic Processes

**Figure 3:**
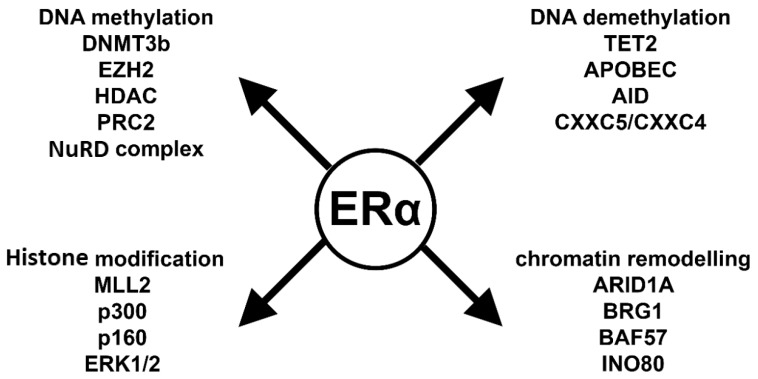
ERα-related epigenetic interactome. DNA methylation: DNMT3B: DNA methyltransferase 3b; EZH2: zeste homolog 2; HDAC: histone deacetylase; PRC2: polycomb complex 2; NuRD complex: nucleosome remodeling deacetylase. DNA demethylation: TET2: ten-eleven translocation enzyme 2; APOBEC: apolipoprotein B mRNA editing enzyme; AID: activation induced cytidine deaminase; CXXC5/CXXC4: CXXC-type zinc finger protein 5 and 4. Histone modification: MLL2: mixed lineage leukemia gene 2; p300: E1A Binding Protein 300; p160: steroid receptor coactivator; ERK1/2: extracellular signal-regulated kinase 1/2. Chromatin remodeling: ARID1A: AT-Rich Interaction Domain 1A; BRG1: actin dependent regulator of chromatin, subfamily a, member 4; BAF57: Actin Dependent Regulator of Chromatin, Subfamily E, Member 1; INO80: chromatin remodeling INO80 complex.

As demonstrated above, the mechanisms of E2-induced epigenetic processes are extremely complex. Depending on the reaction partner, ERα can repress or promote gene expression changes. To provide a better understanding, we summarize the key players of these processes in Figure 3 and below.

Liganded ERα plays a pivotal role in the CpG island methylation process, inducing the expression of DNMT3b [28,32]. Moreover, ERα recruits co-repressor proteins such as HDAC1 and MTA1 from the NurD complex and EZH2 from the PRC2 complex. All together these molecules activate DNMT3b and cause CpG island methylation that represents a repressive mark on the DNA [29]. In summary, ERα can effectively repress gene transcription via DNA methylation [29,96].

E2 also plays a critical role in both passive and active demethylation processes. Liganded ERα blocks DNMT1 expression; therefore, the newly replicated DNA lacks methylation marks. It forms complexes with active demethylation proteins such as TET2, CXXC4, CXXC5, APOBEC, AID and TDG [61,62,63,66]. These proteins first modify and later remove the repressive methyl mark from the DNA and thereby promote gene expression.

ERs also interact with histone modifying enzymes. ERα interacts with histone acetyl transferases such as p160, p300 and MLL2 [77,78,83]. These proteins covalently modify the histone lysine residues and change transcriptional activity. H3K4 modifications can be either acetylation or methylation, which are activation marks. However, the monomethylation, dimethylation or trimethylation of H3K27 is a repressive mark.

The regulation of gene activity is not possible without changing the accessibility of chromatin to the gene transcription machinery. ERα interacts with ATP-dependent chromatin remodelers such as SWI/SNF complex. The BRG1 and BAF57 proteins have transcription initiation roles and interact with ERα, similarly to the INO80 protein complex [88,89,90,97]. ARID1A is a key protein in the basal cell transition of breast cancer, and it plays a pivotal role in ERα-induced gene transcription [98].

## 7. The Physiological and Pathophysiological Relevance of E2-Induced Epigenetic Mechanisms

The critical question related to the E2-induced epigenetic mechanism is that of identifying the physiological or pathophysiological relevance of the observed changes in the DNA. E2 initiates a wide range of epigenetic changes during embryonic development, in the brain and in breast cancer [60,78,99].

Histone acetylation plays a pivotal role in memory formation in both female and male mice. During memory consolidation, ERs indirectly activate acetyl transferases via the ERK1/2 signaling pathway [32,33,75,100,101]. Importantly, ERα-induced ERK1/2-mediated H3 histone acetylation enhanced memory in the novel object recognition test in female mice. In male mice, even though E2 also stimulates memory consolidation in novel object recognition, the molecular mechanism is not known [75].

Both ERα and ERβ play a complex regulatory role in spermatogenesis. ERα represses the expression of HDAC, which leads to hyperacetylation and, consequently, aberrant histone methylation. ERβ represses all DNMTs, which leads to changes in methylation patterns. Accordingly, the number of methylated histones decreases differently in the testis [74]. Importantly, E2-induced epigenetic defects affect spermatogenesis and are likely to play a critical role in the mechanism of E2-induced infertility [74].

Liganded ER contributes to the silencing of the Ephx2 gene via promoter CpG island methylation. This inhibition contributes to increased levels of the cardioprotective substance, epoxyeicosatrienoic acid, providing a possible explanation for the lowered risk of cardiovascular diseases in women [96].

In breast cancer, E2 silences genes via the methylation of LCN2 and IFI27 [29], playing an important role in the luminal differentiation of cancer cells [29]. DNA demethylation predominantly induces the activation of gene transcription in breast cancers. ERα-positive breast cancer cells are hypomethylated compared to ERα-negative breast cancers [27]. ERα actively demethylates cytosine residues and therefore activates the transcription [61,66] of genes such as APOBEC3B and LCN2 [68]. Targeting these genes could provide a possible platform for the development of future therapies for breast cancer. The chromatin remodeling complexes are also exquisite players in the development of breast cancer. For instance, ARID1A, as a member of the SWI/SNF chromatin remodeling complex, interacts with ERα at the ERE sites in the genome and prevents the luminal cells from transitioning into basal cells [98]. By contrast, the genetic deletion of ARID1A blocks the binding of ERα to the DNA, which promotes basal cell development [98]. Together, these results suggest that the interaction between ERα and ARID1A may provide an effective platform for maintaining the endocrine therapeutic response in ERα-positive breast cancer.

The changes in the epigenetic landscape in postmenopausal age influence the chronological age. For instance, woman whose blood has more methylated DNA than expected may experience an acceleration of senescence [102,103]. Although the decreased level of E2 is responsible for a woman’s body changes during menopause, the role of the E2 in elevated DNA methylation capacity in postmenopausal age is uncertain. Accordingly, further investigations are needed to explore the impact of E2 on epigenetic changes during aging.

## 8. Conclusions

Taken together, E2 as a key player in epigenetic mechanisms, playing a pivotal role in breast cancer development and spermatogenesis as well as in memory formation. Understanding the E2-related epigenetic processes provides a novel perspective in E2-induced physiological and pathophysiological mechanisms. Furthermore, investigating the interactions between liganded ERα and epigenetic modifying proteins will aid in the identification of new diagnostic and therapeutic targets. However, more research into the mechanism of action and role of E2 in the epigenetic processes related to physiological and pathophysiological situations is warranted.

## Abbreviations

5caC	5-carboxy-methyl cytosine
5fmC	5-formyl-methyl cytosine
5hmC	5-hydroxy-methyl cytosine
5hmU	5-hydroxymethyl-uracyl
5mC	5-methylcytosine
ARID1A	AT-Rich Interaction Domain 1A
ARID1B	AT-Rich Interaction Domain 1B
AID	activation induced cytidine deaminase
APOBEC	apolipoprotein B mRNA editing enzyme
BER	base excision repair complex
coREST	REST corepressor 1
CpG	cysteine and guanin rich region
CXXC	CXXC-type zinc finger protein
CYP1A1	cytochrome P450
DNMT	DNA methyltransferase
DNMT1	DNA methyltransferase 1
DNMT3a	DNA methyltransferase 3a
DNMT3B	DNA methyltransferase 3b
DNMT3l	DNA methyltransferase 3l
E2	17β -estradiol
EET	epoxyeicosatrienoic acid (EET)
Ephx2	epoxide hydrolase 2 gene
ERE	estrogen responsive elements
ERK1/2	extracellular signal-regulated kinase ½
ER	estrogen receptor
ERβ	estrogen receptor β
ERα	estrogen receptor α
EZH2	zeste homolog 2
H3	histone 3
H3K4	histone 3 lysine 4
H3K27	histone 3 lysine 27
H3K36	histone 3 lysine 36
HDAC1	histone deacetylase
IFI27	interferon α inducible protein 27
LCN2	lipocalin 2 gene
MLL1-3	mixed lineage leukemia genes 1-3
MTA1-3	metastasis associated factors 1-3
NRIP1	nuclear receptor interacting protein
NurD	nucleosome remodeling deacetylase
OCT4	octamer binding transcription factor
p300	E1A Binding Protein 300
PgR	progesterone receptor
PRC2	polycomb complex 2
REA	repressor of estrogen activity
RSK4	ribosomal protein kinase
SAM	S-adenyl methionine
SOX2	sex determining region Y
SRC	steroid receptor coactivator
TDG	thymine DNA glycosylase
TET	ten-eleven translocation enzyme
TRPS1	transcriptional repressor GATA binding 1
